# Strain-promoted sydnone bicyclo-[6.1.0]-nonyne cycloaddition†
†Electronic supplementary information (ESI) available: Full experimental details, ^1^H/^13^C NMR spectral data, protein synthesis and purification. See DOI: 10.1039/c3sc53332h



**DOI:** 10.1039/c3sc53332h

**Published:** 2014-02-05

**Authors:** Stephen Wallace, Jason W. Chin

**Affiliations:** a Medical Research Council Laboratory of Molecular Biology , Francis Crick Avenue, Cambridge Biomedical Campus , Cambridge , CB2 0QH , UK . Email: chin@mrc-lmb.cam.ac.uk ; http://www.2.mrc-lmb.cam.ac.uk/ccsb/

## Abstract

The discovery and exploration of bioorthogonal chemical reactions and the biosynthetic incorporation of their components into biomolecules for specific labelling is an important challenge. Here we describe the reaction of a phenyl sydnone 1,3-dipole with a bicyclononyne dipolarophile. This strain-promoted reaction proceeds without transition metal catalysis in aqueous buffer, at physiological temperature, and pressure with a rate comparable to that of other bioorthogonal reactions. We demonstrate the quantitative and specific labelling of a genetically encoded bicyclononyne with a sydnone fluorophore conjugate, demonstrating the utility of this approach for bioorthogonal protein labelling.

## Abstract 2

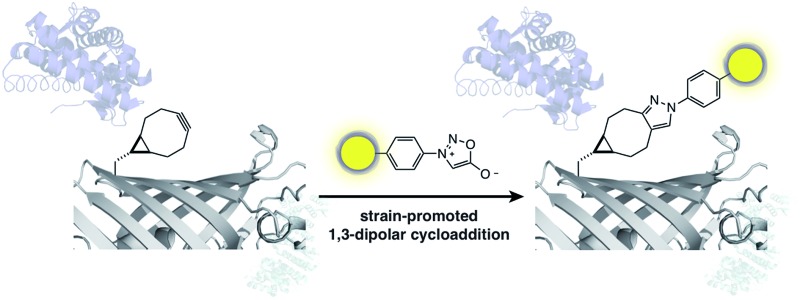
We report the strain-promoted sydnone bicyclo-[6.1.0]-nonyne cycloaddition and demonstrate that this bioorthogonal reaction enables site-specific protein labelling.

## Introduction

The discovery and exploration of bioorthogonal chemical reactions and the biosynthetic incorporation of their components into biomolecules for specific labelling is an important challenge. A variety of reactions have been described, including: the reactions of aldehydes and ketones with alpha-effected nucleophiles,^1^ traceless Staudinger ligations,^2^ copper-catalysed terminal azide–alkyne cycloadditions,^3^ strain-promoted azide–alkyne cycloadditions,^4^ strain-promoted alkyne–nitrone cycloadditions,^5^ variants of ruthenium-catalysed cross-metathesis^6^ and palladium-catalysed cross-couplings,^7^ photo-click chemistry,^8^ cyanobenzothiazole condensations with 1,2-aminothiols^9^ and inverse electron-demand Diels–Alder reactions between strained alkenes or alkynes and tetrazines.^10^ The rate constants for these reactions span approximately 10 orders of magnitude, and range from 10^–5^ M^–1^ s^–1^ to 10^5^ M^–1^ s^–1^.^11^ While Hüisgen's seminal work on azide–alkyne 1,3-dipolar cycloadditions was crucial to the subsequent development of bioorthogonal chemistry and “click” chemistry,^12^ his concurrent work on the dipolar cycloaddition chemistry of aryl sydnones has received comparatively little attention.^13^ Sydnones typify a small class of mesoionic 5-membered heterocycles first discovered by Earl *et al.* in 1935,^14^ and can be considered as cyclic 1,5-dipolar aromatic azomethine imines.

Sydnones undergo thermal [3 + 2] cycloaddition with a range of dipolarophiles to afford substituted pyrazole products. Hüisgen reported that *N*-phenylsydnone undergoes cycloaddition with acetylene after 25 h at 170 °C to afford *N*-phenyl pyrazole in 75% isolated yield. Very recently, a copper-catalyzed sydnone–alkyne cycloaddition reaction using a Cu–phenanthroline catalyst was reported.^15^ This complex catalyzes the cycloaddition of phenyl sydnone and a range of mono-substituted alkynes (20 mol% catalyst, 11 : 9 ^*t*^BuOH–H_2_O, triethanolamine, sodium ascorbate) in up to 99% yield after 16 h at 60 °C. Additionally, lysine residues in a bovine serum albumin (BSA) sample were reacted with a succinimidyl-activated sydnone derivative *in vitro*. The resulting protein adduct(s) reacted with a dansyl-conjugated propargylamine in the presence of CuSO_4_/ligand (pH 8.0) at 37 °C after 16 h to afford a mixture of protein species that are labelled (non-quantitatively) to various extents. Based on these precedents and the precedent for accelerating the azide–alkyne and alkyne–nitrone cycloaddition reaction by using pre-installed conformational strain^4,5^ we decided to investigate the potential reaction of a strained alkyne with phenyl sydnones for the site-specific labelling of proteins.

Here we describe the reaction of a phenyl sydnone 1,3-dipole with a bicyclononyne dipolarophile. This strain-promoted reaction proceeds without transition metal catalysis in aqueous buffer, at physiological temperature and pressure with a rate comparable to that of other useful bioorthogonal reactions. We demonstrate the quantitative and specific labelling of a genetically encoded bicyclononyne with a sydnone fluorophore conjugate, demonstrating the utility of this approach for bioorthogonal protein labelling.

## Results and discussion

We first synthesized phenyl sydnone (**1**) and *exo*-((1*R*,8*S*)-bicyclo[6.1.0]non-4-yn-9-yl)methanol (BCN, **2**). BCN was synthesized in 4 steps *via* cyclopropanation of 1,5-cyclooctadiene. Phenyl sydnone was synthesized in 2 steps from *N*-phenyl glycine *via N*-nitrosylation and intramolecular cyclization using acetic anhydride. The synthesis of **1** requires no column chromatography as the products of each synthetic step can be isolated by simple recrystallization, and **1** is a stable solid at room temperature.

**Scheme 1:**
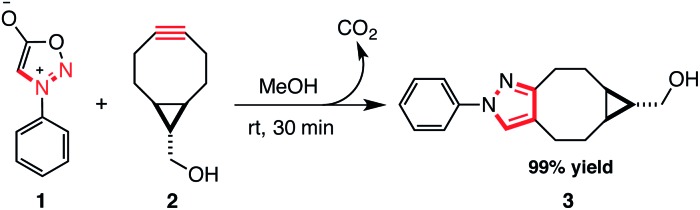
The strain-promoted 1,3-dipolar cycloaddition of phenyl sydnone **1** with BCN **2**.

Next, we investigated the potential reaction of aryl sydnones with BCN. When equimolar amounts of phenyl sydnone and BCN were combined in methanol at room temperature we observed clean cycloaddition to the corresponding pyrazole in 30 min, with an isolated yield of 99%. This reaction appears to be much faster than the copper-catalysed cycloaddition of phenyl sydnones to terminal alkynes, which requires 20 mol% of a Cu^I^ catalyst and 16 h at 60 °C to achieve comparable yields.^15^ We suggest that the reaction proceeds *via* initial suprafacial [3 + 2] cycloaddition to afford a diaza-[2.2.1]-bicyclic lactone, which spontaneously undergoes a cycloreversion with the extrusion of carbon dioxide to afford a cyclooctane-fused *N*-phenyl pyrazole (see S1†) (Scheme 1).^13^

The rate constant for the cycloaddition was determined under *pseudo*-first order conditions by following the exponential decay in phenyl sydnone absorbance at 310 nm over time upon reaction with a 10–80 fold excess of BCN in 55 : 45 MeOH–H_2_O. The determined rate constant was 0.054 M^–1^ s^–1^ (±0.00067 M^–1^ s^–1^) at 21 °C, which is comparable to other strain-promoted [3 + 2] cycloadditions with demonstrated utility.^4^ The rate constant for the sydnone–BCN cycloaddition is greater than the rate constant reported for Staudinger ligations or uncatalysed ketone condensations with alpha-effected nucleophiles, and comparable to rate constants for some cross-metathesis reactions or strain-promoted azide–alkyne cycloadditions. However, the reaction is slower than the cyanobenzothiazole condensation with 1,2-aminothiols, strain-promoted alkyne–nitrone cycloadditions, photo-click chemistry, copper-catalysed azide–alkyne cycloadditions or inverse electron-demand Diels–Alder reactions between tetrazines and strained alkenes and alkynes.^11a,11b^

**Fig. 1:**
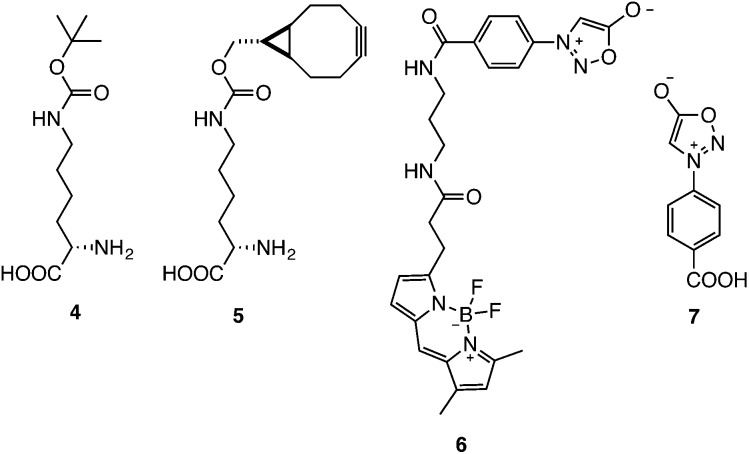
The structures of the unnatural amino acids **4**/**5** and the sydnone derivatives **6**/**7** used in this study.

To demonstrate the biocompatibility of the reaction a BCN-containing unnatural amino acid (BCNK, **5**) was site-specifically incorporated, *via* genetic code expansion, into a recombinant super-folder GFP protein.^16^ Labelling of the encoded unnatural amino acid with phenyl sydnone **1** and phenyl sydnone derived fluorophore **6** was characterised (Fig. 1).

**Fig. 2:**
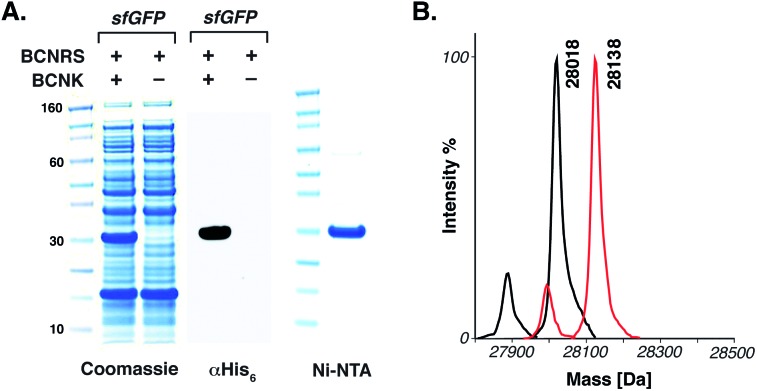
The genetic incorporation of **5** in *E. coli*. (A) Amino acid dependent overexpression of *sf*GFP-**5**_150_. Protein was detected in lysate using an anti-His_6_ antibody and by Coomassie staining. *sf*GFP-**5**_150_ was purified using Ni–NTA beads. (B) ESI-MS data for amino acid incorporation and quantitative site-specific labelling of *sf*GFP-**5**_150_ with *N*-phenyl sydnone **2**. For *sf*GFP-**5**_150_: calculated 28 018, found 28 018. For *sf*GFP-**5**_150_ labelled with **1**: calculated 28 136, found 28 138. Minor mass peaks represent proteolysis of the N-terminal methionine (see S4 and S5†).

The BCNRS/*t*RNA_CUA_ pair (a mutant of the orthogonal *Methanosarcina barkeri* pyrrolysyl-*t*RNA synthetase (*Mb*PylRS)/*t*RNA_CUA_ pair that directs the incorporation of **5** containing three mutations in the enzyme's active site (Y271M, L274G and C313A))^10*e*^ and a gene encoding a C-terminally hexahistidine-tagged *sf*GFP containing an amber codon (TAG) at position 150 (*sf*GFP_150TAG_His_6_) were introduced into *E. coli*. Addition of **5** (2 mM) led to the amino acid dependent synthesis of full-length *sf*GFP in good yield (5 mg L^–1^ of culture). Recombinant *sf*GFP bearing **5** at position 150 (*sf*GFP-**5**_150_) was purified using Ni–NTA chromatography and ESI-MS confirmed the genetically-directed incorporation of **5**. To demonstrate the utility of the reaction for site-specific protein labelling we incubated *sf*GFP-**5**_150_ (4 pmol) with 50 eq. of phenyl sydnone **1** in aqueous buffer (20 mM Tris–HCl, 220 mM imidazole, 300 mM NaCl, pH 8.0) at 37 °C. After 6 h we observed a single product, corresponding to quantitative cycloaddition of the sydnone to the encoded BCN by mass spectrometry (Fig. 2).

**Fig. 3:**
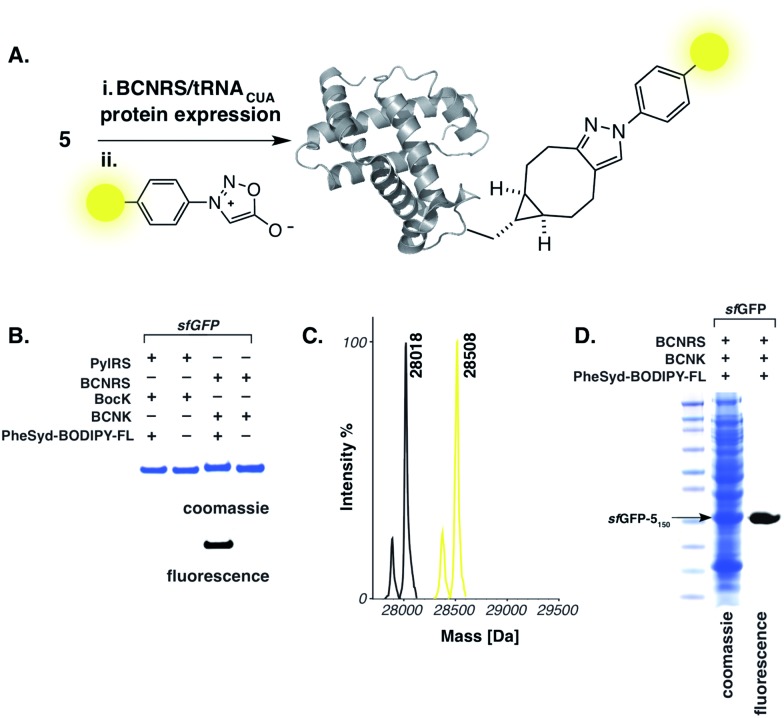
(A) Genetic encoding and fluorogenic labelling of **5** *via* the strain-promoted sydnone–bicyclononyne cycloaddition. (B) Specific labelling of *sf*GFP-**5**_150_ with fluorescent sydnone conjugate **6** using Ni–NTA-purified *sf*GFP-**5**_150_. (C) ESI-MS data for quantitative labelling of *sf*GFP-**5**_150_ with PheSyd–BODIPY-FL **6**. For *sf*GFP-**5**_150_: calculated 28 018, found 28 018. For *sf*GFP-**5**_150_ labelled with **6**: calculated 28 510, found 28 508. The minor mass peaks represent proteolysis of the N-terminal methionine (see S4 and S6†). (D) Specific fluorescent labelling of *sf*GFP-**5**_150_ with **6** in *E. coli* cell lysate.

To further demonstrate the specificity of the reaction for protein labelling and to allow visualization of the cycloaddition product on proteins a fluorescent sydnone–BODIPY conjugate (PheSyd–BODIPY-FL, **6**) was synthesized in three steps from *p*-carboxylphenyl sydnone **7** in 52% overall yield. When 4 pmol of purified *sf*GFP-**5**_150_ was incubated with 0.2 nmol **6** (20 mM Tris–HCl, 220 mM imidazole, 300 mM NaCl, pH 8.0, 37 °C, 6 h) the reaction leads to a mobility shift of the protein in Coomassie-stained SDS-PAGE. Fluorescent imaging reveals that the protein is fluorescently labelled. Control experiments in which BocK (**4**) is incorporated into the protein in place of **5** (*sf*GFP-**4**_150_) demonstrate that the labelling reaction is dependent on the presence of BCNK in the protein. ESI-MS demonstrates quantitative labelling of *sf*GFP-**5**_150_ with **6**. To further demonstrate the specificity of the reaction we carried out labelling in an *E. coli* lysate in which sfGFP-**5**_150_ was present at a comparable level to many *E. coli* proteins. Despite the presence of a number of proteins at comparable levels to *sf*GFP-**5**_150_ we observe clear and selective fluorescent labelling of *sf*GFP-**5**_150_ with **6** (PBS, pH 7.4, 37 °C, 6 h) (Fig. 3).

## Conclusions

In conclusion, we report a new strain-promoted bioorthogonal reaction between *N*-arylated sydnones and bicyclo-[6.1.0]-nonyne (BCN, **2**). The uncatalysed cycloaddition of *N*-phenyl sydnone and BCN proceeds to completion at ambient temperature in organic solvent and in aqueous buffer. We have demonstrated the quantitative site-specific labelling of proteins bearing a genetically incorporated BCN with phenyl sydnone **1** and PheSyd–BODIPY-FL **6** in aqueous buffer at 37 °C and demonstrated that the reaction is chemoselective with respect to the *E. coli* proteome. Future work will explore the scope of this reaction for biomolecule labelling and the effect of substituents on the components of the reaction for tuning its properties *in vivo*.
